# Chloride of Sodium

**Published:** 1868

**Authors:** A. Means

**Affiliations:** Oxford, Ga., Professor of Chemistry in Atlanta Medical College


ATLANTA

### Medical and Surgical Journal.

NEW SERIES.

Vol. IXA. MARCH AND APRIL, 1868. No. 1.

### ORIGINAL COMMUNICATIONS.

### ARTICLE I.

Chloride of Sodium. By A. Means, M. D., of Oxford, Ga., Professor of Chemistry in Atlanta Medical College.

The *Chloride of Sodium* (marine or common salt) is probably found in greater quantities in all *organized* bodies than any other *inorganic* compound, and is the only article in the long list of condiments which is essential to health. Now, the God of Nature has furnished to all beings an instinctive desire or appetite for such nutrient articles as promote their growth and development, secure their preservation, and contribute to their well-being. Nay, more—he has prepared in each an apparatus suited to the digestion, absorption and appropriation of each, and even furnished prehensile appliances adapted to their procurement. Furthermore—such articles, by the order of Divine wisdom and goodness, afford absolute gratification and enjoyment in their use. All these distinctive evidences of *design* are realized in the case of common salt.

It is the plan of Providence, that where there is an extensive or universal demand for elements of nutrition, assimilation, respiration, etc., there will be found an abundant or world-wide supply. *E. g.*—*Oxygen* is prepared for the *whole breathing* world that requires it, whether tenants of the atmosphere, the ocean, lakes or rivers ; and *water* for the healthy maintenance, growth and prosperity of the entire animal and vegetable kingdoms—*both* exciting pleasurable emotions in the animal creation, when its demands are gratified.

A fondness for salt, then, which characterizes our race, has existed in all ages, and pervades all nations, classes and conditions of mankind, whether civilized, semi-civilized, or savage, is a strong indication that animal nature requires it for higher purposes than mere sensual gratification, and that in comformity with the providential economy above named, we should expect to find a liberal and diffusive supply of this valuable *haloid* salt, throughout the globe.

Now, *both* of these postulates are assumed and sustained under the sanctions of *philosophy* and *fact.*

The *first* has been vindicated by the researches of chemico-physiology, and the essentiality of the Chloride of Sodium to the full maintenance of the normal functions of the animal economy thus satisfactorily ascertained. It forms an indispensable constituent of the *blood*, and is borne through every portion of the system, to aid in the production of many of its vital phenomena. It is found in the various secretions—as in tears, in bile, in perspiration, etc., and, indeed, in every solid and fluid of the human body, except *enamel*, to the aggregate of 227 grains; and is constantly undergoing decomposition and waste, which can alone be sufficiently repaired by the quantity taken into the stomach, as condiment. Again; free *hydro-chloric acid* is found in the *gastric juice*, the great agent of digestion; and this is evidently formed from a union of the *chlorine* of the salt with the *hydrogen* of *sulphydric* acid, so liberally evolved in the digestive process, or with the same element (hydrogen) taken away, by elective affinity, from the serous portion of the blood, or from *both.* The other forms of soda, too, found in the circulating mass, as the carbonate, the tri-basic phosphate, etc., are probably owing to the decomposition of the chloride. Nor is it improbable, from Leibig's analysis, that the latter *salt*, in juxtaposition with free lactic acid, in the nucleated cells of the muscular fibrillæ, complete the galvanic arrangement which largely generates the electro-vital currents, essential to the mental and physical phenomena of life.

Again; salt is an *antiseptic*—prevents or corrects a tendency to putrescence, either *within* or *without* the human system, and is known to be destructive to parasitic animals which infest the stomach and alimentary canal, as ascarides, lumbricoides, etc. Hence, those who use little or no salt with their food are very subject to be affected by worms. "The ancient laws of Holland," says Lord Summerville, "ordained men to be kept on bread *alone*, *unmixed* with *salt*, as the severest punishment that could be inflicted upon them in their moist climate. The effect," says he, "was horrible. These wretched criminals are said to have been *devoured* by *worms*, engendered in their own *stomachs*." In the *Medical and Physical Journal*, Dr. Marshall reports the case of a lady "who had a natural aversion to salt, and who was most dreadfully afflicted with worms during the whole of her life." Let children, then, be accustomed to use provisions properly imbued with salt, and their mothers will have less need to resort to vermifuges.

It is also a *tonic*, and, in moderate quantities, acts as a stimulus "to the mucous membranes, the absorbent vessels, and glands." Where there is a deficiency of salt in the blood, that fluid assumes "a dark or blackish hue," and is often accompanied by intractable diseases. This state of things is strikingly exemplified in Asiatic cholera.

It is not, therefore, true, as has been sometimes asserted, that human aliment, unseasoned by salt, as in the savage state, is favorable to long life; for the most ancient and reliable statistics show that longevity is *greatest*, and the highest average duration of life "occurs in countries where wealth, commerce and civilization are most universally diffused," and where the habitual use of salt among the population is consequently without exception; while, on the contrary, the average is *lowest* where poverty, ignorance and despotic government prevail, and which embraces every form of native barbarism, as well the American aborigines as others. *E. g.*—In enlightened and prosperous England, the rate of mortality is only 2¼ per cent. of the population; whereas, amid the millions of the ignorant and degraded serfs of autocratic Russia, the average, throughout the empire, is 3½ per cent.

It is true, however, that savages who know not the use of salt may pass through an ordinary life without it, but never illustrate the perfection of mental, moral and physical manhood. In such cases, the utmost that can be said is, that nature makes the most of her limited resources, as in the case of vegetarians, who ignore the use of animal flesh, so rich in nitrogen, the base of muscle, and indicated as an appropriate diet, as well by the structure and conformation of the human teeth as by the universal proclivity for that class of food—dependent, in the meantime, for the scanty supply of nitrogen which they receive upon the plants and fruits which they consume.

Nor let it be plausibly urged, that because we have phosphorus, sulphur, potassa, lime, etc., among the constituents of the human body, that we should *eat these*, as well as salt, in our daily food: First—Because, as independent chemical agents, they would act as corrosives, poison, or medicines, upon the coats of the stomach and intestines. Second—Because, for the slow metamorphosis of the brain, bone, muscle, and other tissues, as well as for the elaboration of milk, bile, saliva, and other secretion—these articles, in a *combined* state, and harmless, are taken into the system in sufficient quantities by the common aliment—vegetable albumen, fibrin and casein, found in potatoes, beans, peas, oily seeds, etc., containing each about 20 per cent. of sulphur and phosphorus; while the gluten of wheat supplies nearly 16 per cent. of nitrogen; other cereals, a less quantity; and the fibrin of animal muscle alone, used as a food, about 15.75 per cent. So, too, of potassa, lime, magnesia, etc.; for the human system boasts no element in its composition which is not found *in*, and obtained *from*, the external world.

But secondly: Nature indicates the great necessity for this saline condiment, by the immense and widespread supply which she affords, in solid and fluid form. A glance alone can be taken of this extensive field.

*Rock* salt, in vast deposits, is found in Prussia, especially at Wieliczka—in the Tyrol, or crown-lands of Austria—in Bavaria, Saltzburg, Hungary, France, Switzerland, Spain, England,

Ireland and Russia, and in both continents of America, to name no other regions; while the supply from lakes, lagoons, salt springs and sea-water, is inexhaustible. Indeed, excepting Norway, Denmark and Holland, every country in Europe has its own domestic sources, and *these* kingdoms are regularly supplied by commercial interconrse. Even Asia and Africa have not been forgotten, The *former* is profusely furnished with saline incrustation, or salt lakes and wells, as in Tartary, Armenia, at lake Ooroomiah, (ninety miles long and thirty broad, charged with brine containing 18 per cent. of pure salt, crystalizing, for miles together, a foot thick upon its surface,) and in China and Japan. The latter (Africa) has large deposits in the desert of Saharah, whose yield is a chief source of traffic with Soodan. Two mountains of salt are here found, and the lake Lagres has its surface covered with beautiful white crystalization, sometimes too feet thick. In Central Africa, it is a cheap article of commerce, and regarded so much of a luxury that children eat it like sugar. Mungo Park, the great African travrler, experienced great distress from being, in one locality, deprived of it. And here allow us to remark that the discovery, use, and commercial importance of this pleasurable and health ful relish, among a rude and unenlightened people, is perhaps entirely attributable to the European and American adventurers who have, for half a century, occupied the coasts and interior of Central Africa, and whose habits, customs, and forms of religion, have been gradually molding these "sons of the desert." And the age *may* come when Nature's vast reservoirs of salt, in these dark regions, may be in demand for the native population. As if foreshadowing this devoutly to be wished result, the French, English, Portuguese and Danes, the Northerlands and the United States, have numerous settlements, forts, castles and factories there, and their farmers, manufacturers, mechanics, merchants and missionaries are leaving their impress upon popular manners wherever they have access.

But why individualize further? The intellect, the labor and the capital of the *world* have been willingly and extravagantly taxed, and Earth's ample store-houses have liberally responded to furnish in sufficient quantities this great, indispensable sweetener of life. As a synopsis of the whole, then: England turns out 5,000,000 of *tons annually;* United State, say about 500,-000; while the English and Dutch West Indies, Mexico, Asia, Africa and other countries supply in the aggregate, say 500,000; thus making the entire annual products of the world *six millions of tons* (6,000,000,) or *twelve thousand millions of pounds* (12,000,000,000.)

But the instincts of quadrupeds and birds lead them to seek this vivifying chemical alterative in their native state; and a benign Providence, besides the amount of its constituents which they obtain through their ordinary food, has spread out the salt plains for the buffalo and the deer, the elk and the wild ox of the forest, and salt lakes and springs for the fowls of Heaven. Indeed, says an able writer, "neither animals nor plants will thrive when totally deprived of salt," though too great a quantity may prove deleterious, as may any other article of diet. Nor must we forbear to remark that salt has been honored in the ***Bible*** record, and made significantly typical of holier things, among the Hebrews. All the sacrifices of the temple were seasoned with it; new-born babes were to be rubbed with it; and Elijah employed it to correct and "sweeten" the fountain of Jericho. It is also mentioned as one of the things most necessary to life. And to this day, among the inhabitants of the East, it is used as a symbol of *perpetuity*, *incorruption and hospitality.*

In conclusion, then: while you cannot but regard this as rather a tedious and "*salty*" dissertation upon so common-place a subject, yet you will perceive that your physiological views are, in the opinion of the writer, fully sustained by the philosophy and the facts in the case.*

* This article was written in compliance with the request of Dr. Street, of Tennessee, for Prof. Means' views on this subject.

